# Adipokines in atopic dermatitis: the link between obesity and atopic dermatitis

**DOI:** 10.1186/s12944-024-02009-z

**Published:** 2024-01-23

**Authors:** Shiyun Zhang, Bingjie Zhang, Yuehua Liu, Li Li

**Affiliations:** 1 Eight-year Medical Doctor Program, Chinese Academy of Medical Sciences & Peking Union Medical College, Beijing, China; 2 Department of Dermatology, State Key Laboratory of Complex Severe and Rare Diseases, Peking Union Medical College Hospital, Chinese Academy of Medical Science and Peking Union Medical College, National Clinical Research Center for Dermatologic and Immunologic Diseases, Beijing, China No. 1 Shuaifuyuan, 100730

**Keywords:** Adipokines, Atopic dermatitis, Obesity, Precise therapy

## Abstract

Atopic dermatitis (AD) is a chronic skin condition with intense pruritus, eczema, and dry skin. The recurrent intense pruritus and numerous complications in patients with AD can profoundly affect their quality of life. Obesity is one of its comorbidities that has been confirmed to be the hazard factor of AD and also worsen its severity. Nevertheless, the specific mechanisms that explain the connection between obesity and AD remain incompletely recognized. Recent studies have built hopes on various adipokines to explain this connection. Adipokines, which are disturbed by an obese state, may lead to immune system imbalances in people with AD and promote the development of the disease. This review focuses on the abnormal expression patterns of adipokines in patients with AD and their potential regulatory molecular mechanisms associated with AD. The connection between AD and obesity is elucidated through the involvement of adipokines. This conduces to the in-depth exploration of AD pathogenesis and provides a new perspective to develop therapeutic targets.

## Introduction

Atopic dermatitis (AD) is a common, relapsing skin condition that is influenced by hereditary and immunological factors. The global occurrence of AD is estimated to be around 15%–20% among children and 10% among adults [1]. The primary clinical manifestation of AD is eczema-like lesions on xerotic skin accompanied by severe pruritus. Besides skin lesions, individuals with AD may concurrently exhibit food allergies, allergic rhinitis, conjunctivitis, and asthma. Over half of the AD patients develop the condition in infancy or early childhood. Recommended treatment for mild to moderate AD encompasses general skin protective strategies, topical glucocorticosteroids, calcineurin inhibitors, and phototherapy. Systemic immunosuppression and novel biological therapies may be viable treatments for patients with severe AD [2, 3]. Severe pruritus and associated complications, such as infections, markedly decrease the patient’s quality of life and place a substantial cost on society. In 2017, AD had the highest rate of disability-adjusted life-years among all dermatological diseases, after adjusting for age [4].

AD has been associated with obesity as a risk factor [5]. Research on their shared mechanisms has focused on obesity-related adipokines. Adipokines are recognized as being fundamental to obesity development, and their abnormalities have been extensively observed in patients with AD. Adipokines are regulatory peptides generated by the adipose tissue. Their regulatory functions govern several physiological activities, including energy metabolism, inflammatory response, and immunomodulation. Extensive research has been initiated to examine the involvement of adipokines in AD pathogenesis, indicating a potential connection between AD and obesity.

Investigations on the involvement of adipokines in AD are expected to drive subsequent research on targeted therapeutic approaches. Given the heterogeneity and chronic nature of AD, future therapies are likely to be oriented on precision medicine with enhanced efficacy and minimal side effects. Nonetheless, approved biologic therapies such as dupilumab may not suffice for all patients [6]. Recent research indicates that overweight and obesity may predict a suboptimal response to dupilumab treatment [7, 8]. Adipokines hold potential as novel, precise therapeutic targets. Previous studies have explored therapeutics targeting adipokines in various diseases. In a recent study on obesity, significant improvements in weight and glucose tolerance were observed in obese rats following the administration of a monoclonal leptin antibody [9]. In a U.S.-based study, adipocyte-targeted chitosan nanomicelles were used for adiponectin gene delivery to adipocytes. This intervention normalized serum adiponectin levels and reversed insulin resistance in obese diabetic rats following a single dose [10]. Leptin peptide and adiponectin receptor agonists led to the suppression of tumor growth in breast cancer [11].

Therefore, a comprehensive review of the most recent studies about the contribution of different adipokines in AD was performed to explore their potential to link AD and obesity, improving the understanding of AD pathogenesis and identifying potential targets for future treatment strategies.

## Connection between atopic dermatitis and obesity

Numerous studies have shown the connection between AD and obesity [12]. As recorded, obesity could contribute to the more frequent occurrence of AD as well as exacerbate symptoms [13–17]. Moreover, there is a higher prevalence of obesity in AD patients in comparison to non-AD individuals [17]. Significantly, the influence of obesity was markedly evident in children who became obese before age five, implying that early childhood obesity might raise children's chances of AD development [17]. A large-scale study involving a sample size of 2090 adult patients has shown a clear link between the occurrence of AD and obesity [15]. In addition to clinical studies, a U.S. survey investigated the impact of obesity on AD using two different AD mice models. The study demonstrated a substantial 2–fourfold augmentation in ear thickness in obese mice, indicating a significant increase in the inflammatory response of AD. Persistent inflammation due to obesity resulted in exacerbated AD severity even after the obese mice achieved a weight comparable to that of the control group [18]. In a capsaicin-induced AD rat model, rats demonstrating substantial weight gain presented with more severe skin lesions [19]. In patients with AD, weight loss significantly decreased AD severity [20].

Furthermore, geographical and sex-related variables may affect the connection between AD and obesity. Research has indicated an increase in AD prevalence among obese individuals in Asia and North America [14]. Some studies suggested that only in females a notable association between these two diseases was identified [21, 22]. However, another study has established a substantial correlation between obesity and AD in both males and females, exhibiting similar crude odds ratios (ORs) for men (3.1, 95% CI: 1.4, 7.2) and women (3.2, 95% CI: 1.4, 7.3) [16]. The discrepancies in findings may be ascribed to using body mass index (BMI) in their research, suggesting that the impact of obesity on AD may primarily stem from the effect of adiposity [23].

Despite the established correlation between AD and obesity, the exact molecular mechanism underpinning this association remains unclear. The chronic inflammatory condition of adipose tissue in obese individuals can disturb the immune system function and inflammatory balance, thereby escalating the risk of AD [24]. In addition to its traditional function in energy metabolism, adipose tissue is now being acknowledged as a part of the endocrine system [25]. Adipose tissue comprises multiple cells such as adipose precursor cells and all of them are capable of synthesizing and secreting diverse active compounds. The active substances predominantly comprise adipokines and also encompass cytokines These typically perform immunomodulatory functions and regulate inflammation. The dysregulation of adipokines is hypothesized to contribute to the pathophysiology of obesity with chronic inflammation, [26]. Adipokine dysfunction, prevalent in AD, could be pivotal for its development and may establish a link between obesity and AD.

## Immune and inflammatory mechanism of atopic dermatitis

The etiology of AD is not entirely understood. The process is intricate and affected by both external and internal influences. Exposure to allergens (pollen, dust mites, milk, and seasonal or temperature fluctuations) can trigger AD. Impaired skin barrier function, genetic predisposition, skin microbiome, and mood disturbances are significant predisposing factors for AD [27]. Each of these elements can initiate aberrant inflammatory and immune responses via distinct pathways [28]. Present AD models emphasize the interplay among epidermal barrier defects, skin microbiota, and immune dysregulation [29].

**Fig. 1:**
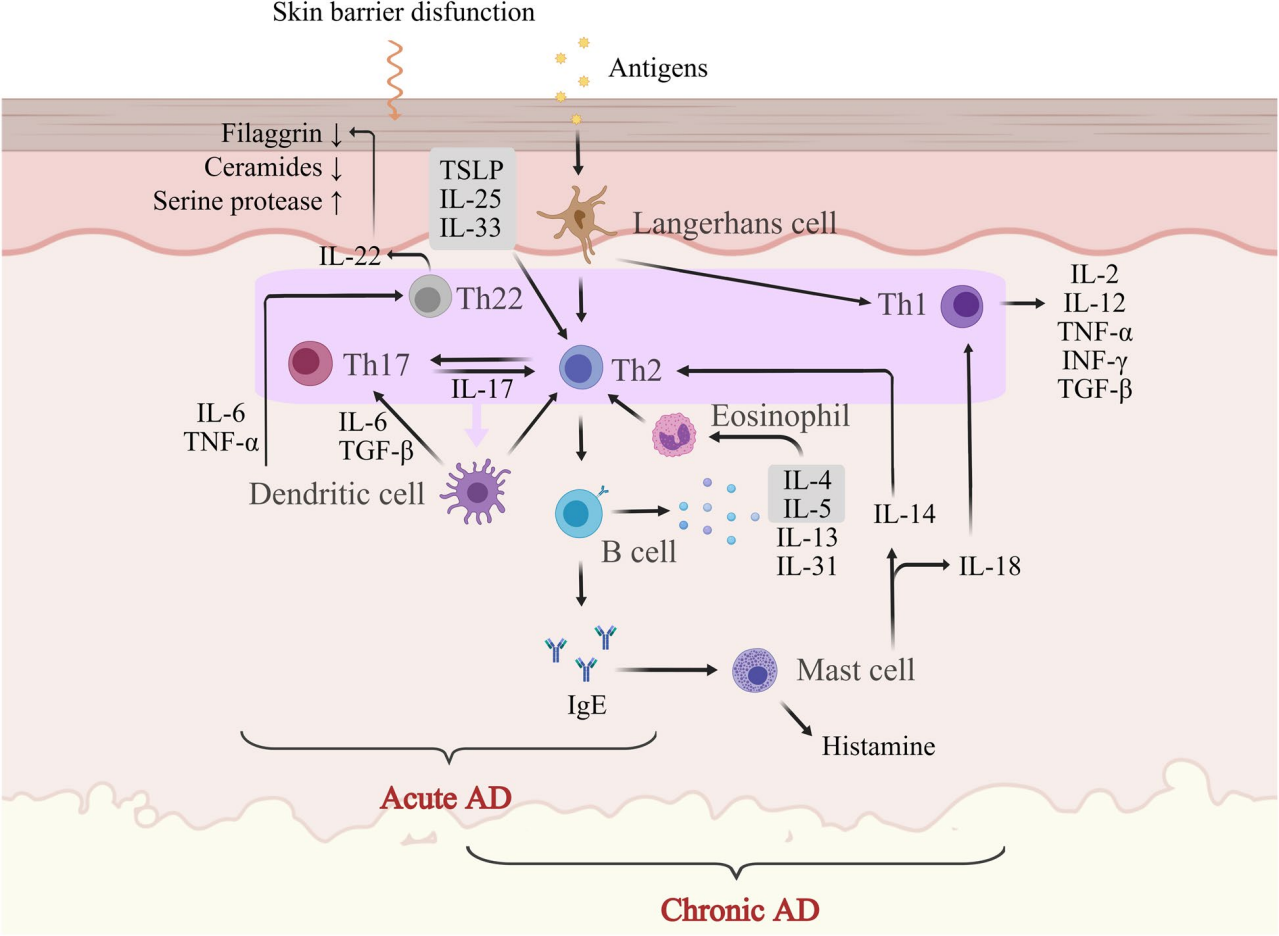
Immune and inflammatory pathogenesis of AD in skin. The overall processes underlying the development of atopic dermatitis in the skin are characterized by an imbalance in T-cell activity and impaired functioning of cytokines. AD, atopic dermatitis; Th, helper T; TSLP, thymic stromal lymphopoietin; IL, interleukin; TNF, tumor necrosis factor; INF, interferon; TGF, transforming growth factor

AD is commonly characterized as a T-helper (Th) 2-mediated inflammatory skin disease, with increased interleukin (IL)-4 and IL-13. Patients with AD have indeed been documented to exhibit phase-dependent differences in immunological dysfunction. Th2 and Th22 immune responses are believed to be chiefly activated during the acute phase of AD, with a comparatively reduced induction of Th17 markers. Patients with chronic AD exhibit an amplified Th1 response in addition to upregulated Th2 and Th22 responses [30–32] (Fig. 1).

In patients with AD, hyperactive Th2 lymphocytes produce IL-4, IL-5, IL-13, and IL-31. These cytokines stimulate the proliferation of eosinophils and trigger the release of immunoglobulin E (IgE) by B lymphocytes via the Janus kinase (JAK) pathway [33, 34]. Elevated IL-4 and IL-13 levels contribute to a reduction in filaggrin (FLG), a protein integral to the preservation of epidermal barrier integrity. This consequently triggers the Th2 response, stimulating keratinocytes to generate proinflammatory cytokines. IL-13 stimulates sensory neurons and mediates the development of chronic itch [35]. Th1 cells exacerbate T-cell skin infiltration by producing substantial amounts of tumor necrosis factor (TNF)-β, and (IFN) -γ. Th17 cells secret IL-17 and IL-22, and IL-22 is also produced by Th22 cells. Recent research has reported that IL-17 was upregulated in a specific group of individuals with AD, although its correlation with disease activity was not as significant as that of IL-22. Increased secretion of IL-22 was particularly noticeable in AD [36].

The differentiation of T lymphocytes in AD patients may be influenced by ethnicity, regardless of the stage of AD. Activations of Th1 and Th17 are reported to be absent in black individuals [37]. Conversely, Asian patients with AD exhibit heightened activation of Th2 and Th17 pathways in their skin tissues, as compared to American and European patients. Furthermore, Th2 and Th22 pathways are typically present in Asian patients with AD [38]. Onset age may also influence T-cell differentiation. Adult-onset AD exhibits a more pronounced Th1-skewing, compared with the notable upregulation of Th17 response in pediatric-onset AD [39–42]. Children with AD exhibit similar or heightened levels of Th2 cytokines and overexpression of Th17-related markers, akin to adult patients with psoriasis [43].

## Role of adipokines in atopic dermatitis

### Materials and methods

To review the research, a search was carried out in both PubMed and Embase to look for studies that were published before September 2023. Combinations of the key terms were used for data retrieval, including “atopic dermatitis,” “adipokine,” “leptin,” “adiponectin,” “resistin,” “visfatin,” “dipeptidyl peptidase-4,” “fatty acid binding protein,” “apelin,” and “zinc-α2-glycoprotein.” Studies that reported the expressions of the mentioned adipokines and adipokines-related genetic variations in patients with AD as well as animal and in vitro experiments focused on the molecular mechanism of adipokines in AD were involved. Reviews, conference abstracts, and case reports were excluded.

### Leptin

Leptin, the inaugural adipokine identified, is synthesized by the obese gene. It influences a range of physiological processes by binding to the transmembrane leptin receptor [44–47]. It is primarily secreted by white adipocytes with a small amount from other tissues [48, 49]. Leptin can be disseminated to various body regions via paracrine, autocrine, and exocrine secretion into the circulatory system. It is subsequently involved in the negative feedback loop of the hypothalamus system for energy metabolism [50]. Starvation results in a decrease of leptin, which in turn activates the neural system to increase hunger and reduce energy usage. Conversely, elevated fat accumulation results in increased leptin levels, suppressing feeding behavior and enhancing fat metabolism [51, 52]. With regard to the innate immune system, leptin inhibits neutrophil apoptosis, promotes macrophage phagocytosis, and triggers the release of proinflammatory mediators. It also regulates NK cells and augments the maturation and cytokine production of dendritic cells [24, 53].

Most studies have reported significant alterations in leptin levels in patients with AD. Adults with AD had significantly higher circulating leptin levels in comparison to individuals of normal weight [20]. Moreover, within the AD patient population, individuals with obesity demonstrated higher serum leptin levels than those of standard weight. These findings suggest that leptin dysregulation is present in patients with AD and that obesity aggravates this dysregulation. Similar studies have also reported increased serum leptin levels in adults and children with AD [54, 55]. Lower leptin levels in patients with AD were observed in two studies with certain methodological limitations. One study investigated the circulating leptin levels of breastfeeding women with AD, in which the potential effects of pregnancy should not be neglected [56]. The other study was limited by a small sample size, encompassing only five patients with AD [57]. In this context, AD is hypothesized to induce an elevation in circulating leptin levels.

Regarding the severity of AD, current evidence indicates that there is no significant correlation between the Eczema Area and Severity Index (EASI) or SCORing Atopic Dermatitis Index (SCORAD) and serum leptin concentrations [20, 58, 59]. A single study noted a significant disparity in circulating leptin levels between mild (SCORAD < 40) and severe (SCORAD ≥ 40) AD groups. In some studies, a positive correlation between the levels of leptin and IgE was found in AD patients [20, 54]. Nonetheless, some studies have shown no significant correlation between the two, suggesting that leptin may be implicated in both IgE- and non-IgE-mediated inflammatory mechanisms [55].

Topical glucocorticoid treatment does not seem to affect serum leptin levels in children with AD [60]. This discovery implies that the primary treatment strategy for AD, which involves the use of topical corticosteroids, only ameliorates the local skin condition. Moreover, leptin expression in individuals is influenced by single nucleotide polymorphisms (SNPs). A study connected the GG allele of the LEP gene SNP rs2167270 to the prevalence of AD, potentially elucidating a portion of the genetic susceptibility to AD [61].

Leptin can be crucial for regulating immunological responses. It stimulates the development of Th1 and Th17 cells while suppressing regulatory T (Treg) cell proliferation [53, 62]. Th1 and Th17 cells have been involved in the development of numerous autoimmune diseases, including AD [1]. Dendritic cells (DCs) undergo Th1 polarization upon activation of the Akt and nuclear factor-kappa B (NF-κB) signaling pathway [63]. Leptin reportedly enhances the differentiation of Th17 cells via retinoic acid-related orphan receptor (ROR)γt in systemic lupus erythematosus [64]. Additionally, leptin facilitates the migration and activation of eosinophils, which produce inflammatory cytokines including IL-1 and IL-6, potentially contributing to Th17 differentiation [65].

Furthermore, leptin activates the mammalian target of rapamycin (mTOR), which is responsible for regulating cellular metabolism, and inhibits Treg cells [66]. Given that Th1 and Th17 cell activation is implicated in AD pathogenesis, leptin’s role in AD development may be elucidated based on its impact on Th1 and Th17 responses.

Leptin typically inhibits the Th2 response by reducing IL-4 and IL-10 levels [67]. Nonetheless, recent studies in allergic asthma and rhinitis suggest that leptin upregulates Th2 cells, type 2 cytokines, and type II innate lymphoid cells (ILC2s) via mTORC1, mitogen-activated protein kinase (MAPK), and phosphatidylinositol 3-hydroxykinase (PI3K)/AKT pathway [68–70]. In the type 2-biased inflammatory environment of AD, leptin may facilitate Th2 differentiation, thereby explaining the observed positive association between IgE levels and leptin concentrations in AD research.

### Adiponectin

Adiponectin, first identified in 1995, is a 244-amino acid protein comprising four distinct regions [71]. Adiponectin receptor 1 (AdipoR1) is located in skeletal muscle, while AdipoR2 is mainly in the liver. These have the potential to enhance tissue insulin sensitivity, facilitate glucose absorption, suppress glycogenolysis, and protect cardiomyocytes [72]. In the regulation of inflammation, adiponectin exhibits two contradictory properties. It has an anti-inflammatory effect in obesity while acting as a proinflammatory factor in rheumatoid arthritis and inflammatory bowel disease [73, 74]. Adiponectin expression is reduced in individuals who have obesity and insulin resistance [75].

Research has demonstrated that AD patients had notably lower levels of serum adiponectin compared to healthy individuals [20, 55, 76, 77]. A German pediatric investigation found a significant association between low levels of adiponectin and a higher occurrence of AD [78]. Adiponectin may reduce inflammation in AD. Interestingly, significant differences were observed in adiponectin levels among patients with varying onset ages of AD. Adult-onset AD is associated with higher levels of adiponectin compared to pediatric-onset AD. This suggests that significant anomalies in adiponectin might potentially contribute to early-onset AD [20].

No substantial association between the levels of adiponectin and the severity of AD is observed. Research has indicated that these levels do not correlate with total IgE, EASI, or SCORAD [20, 55, 58, 77]. Patients with AD can be categorized based on their total Immunoglobulin E (IgE) levels in the blood. Those with IgE ≤ 200 kU/L are classified as having intrinsic AD, whereas those with IgE > 200 kU/L are classified as having extrinsic AD. A Korean study involving 64 patients with AD reported increased levels of adiponectin among extrinsic AD patients [59]. This conclusion contradicts another study that found no significant difference in adiponectin between these two groups [20].

Investigating the effects of dupilumab treatment, a study observed that adiponectin levels did not alter in dupilumab-treated individuals who had significant EASI score improvements. [77]. This suggests that the molecular mechanism responsible for adiponectin abnormalities in AD patients may not coincide with the IL-4/13 pathway. Additionally, a study examined the SNP of the ADIPOQ gene, which encodes adiponectin. It was observed that the frequencies of the GG genotypes for both rs2241766 and rs3774261 were elevated in patients with AD [76]. This suggests that the SNPs rs2241766 and rs3774261 are associated with a genetic predisposition to develop AD.

An in vitro experiment revealed a part of the adiponectin signaling pathway in AD. In the AD-like skin equivalent model, increases in specific mRNAs were observed, including IL-8, TNF-α, human beta-defensin 2, and thymic stromal lymphopoietin (TSLP). This pattern is aligned with the aberrant mechanism observed in AD. Additionally, upon exposure to adiponectin, the mRNA expression of the aforementioned inflammatory mediators returned to levels similar to those of the control group. Adiponectin also upregulated differentiation factors (FLG, involucrin, and loricrin) and lipid biosynthetic enzymes [79]. FLG, loricrin, and involucrin are structural proteins that maintain epidermal barrier function and are downregulated in AD [80]. Enzymes involved in lipid biosynthesis facilitate lipid secretion to protect the integrity of the skin barrier. Adiponectin’s function of increasing FLG has been demonstrated in other studies [81, 82]. Based on the in vitro study, adiponectin treatment could potentially benefit patients with AD by restoring the integrity of the epidermal barrier. The report also indicated a reduction of AdipoR in the AD-like model compared with the control group [79]. Further research is required to ascertain whether analogous receptor alterations occur in patients with AD, potentially inhibiting the adiponectin signaling pathway.

Adiponectin’s anti-inflammatory activities result from the inhibition of keratinocytes, macrophages, and Th17 cells [83]. Adiponectin’s effects on Th2 cells remain unclear. Adiponectin induces IL-10 secretion in Tregs and increases IL-4 production [84]. Administration of adiponectin effectively inhibited NF-κB, thereby mitigating the symptoms of obesity-related asthma [85]. Adiponectin deficiency reportedly promotes macrophage infiltration and Th17 development, which is central to psoriasis pathogenesis [86]. Patients with AD show an adiponectin deficiency, which may promote the Th17 response and contribute to AD pathogenesis. Consequently, patients with pediatric-onset and intrinsic AD may exhibit higher adiponectin levels because of their higher Th17 immune activation [43, 87].

### Resistin

The expression of resistin, identified in diverse tissues, varies across different species. In mice, it is predominantly produced by white adipocytes [88]. Peripheral blood mononuclear cells, adipose tissue macrophages, neutrophils, and sebocytes are responsible for their production [89–91]. Resistin influences insulin resistance, exhibits antimicrobial activity, and regulates inflammation. It is typically regarded as a proinflammatory mediators that activate the NF-κB pathway and induce TNF-α, IL-1β, IL-6, and IL-12 secretion. Resistin decreases DC endocytosis and inhibits the production of IL-6 [92, 93]. Resistin treatment made DCs can promote the proliferation of Treg cells [93]. These findings imply that resistin possesses the ability to suppress inflammatory and immunological pathways. Primarily, elevated resistin levels are correlated with obesity, reduced insulin sensitivity, and increased cardiovascular risk [94, 95]. Nonetheless, certain studies present contrasting results, and a definitive correlation between circulating resistin levels and obesity is yet to be established [96].

Numerous studies have determined that patients with AD exhibit lower resistin levels compared with healthy individuals, a factor that is inversely correlated with disease severity [55, 97, 98]. Subgroup analyses revealed that low resistin levels are associated with increased leukocyte counts and a positive family history of AD [98]. These findings propose that resistin functions as an anti-inflammatory mediator in AD. However, an early study involving children demonstrated elevated resistin expression in patients with AD [99]. These conflicting results may be attributed to the bidirectional regulatory function of resistin in inflammation. Genetically, there are variations in the rs3745367 single nucleotide polymorphism (SNP) of the resistin-encoding RETN gene between control and AD groups. The GG genotype is correlated with reduced resistin levels and a positive familial history of AD, whereas the G allele elevates the risk of AD [97, 98].

### Visfatin

Visfatin is also referred to as nicotinamide phosphoribosyltransferase (NAMPT). The protein is predominantly synthesized in visceral fat but is also generated by other adipose tissue. The function is exhibited both intracellularly and extracellularly [100]. In addition to regulating cellular redox potential, oxidative stress, and cell adhesion via the NAD^+^ pathway, the secretion of extracellular visfatin is associated with cancer, cardiovascular disease, metabolic alterations, inflammatory responses, rheumatic diseases, and AIDS [101, 102]. Research has demonstrated that obese individuals have higher levels of visfatin [103].

Adults with AD were observed to have higher visfatin levels [104]. Another study involving children revealed a significant decrease in serum visfatin levels among individuals with AD [99]. Visfatin levels did not vary across the different severities of AD. However, adult-onset patients displayed elevated levels than those who developed AD in infancy [104]. In terms of laboratory indices, visfatin demonstrated a positive correlation with eosinophil counts but not with IgE levels or VAS itch scores. This finding implies that visfatin could potentially contribute to AD progression by impacting eosinophils in the Th2 immune response.

Furthermore, the dysregulation of visfatin in AD patients implicates adipose tissue in the skin. Immunohistochemical staining of lesioned skin in patients with AD revealed increased visfatin expression in the adipose tissue [104]. This implies that elevated levels of circulating visfatin could be produced by subcutaneous adipose tissue instead of visceral fat. Nonetheless, the association between visfatin and AD remains to be elucidated owing to the scarcity of pertinent research and the heterogeneity observed among existing studies.

Visfatin is a protein that can trigger monocytes to secrete TNF, IL-1β, IL-6, and IL-10 by activating several pathways, such as p38, MEK1, and NF-κB [105]. Visfatin also works with IL-7 to enhance the differentiation of B-cells [106]. In AD, some studies suggest that visfatin may cause a Th17 response and influence the secretion of IgE. More investigations are necessary to comprehend the precise role of visfatin in AD.

### Dipeptidyl peptidase-4 (DPP4)/CD26

DPP4/CD26 is synthesized by adipocytes, thereby making it a novel adipokine. DPP4 exists in two forms: membrane-bound and soluble in bodily fluids. It acts as an intrinsic protease, cleaving peptides containing proline or alanine, subsequently influencing a range of associated physiological functions. DPP4 can also activate T cells and regulate inflammatory and immune responses mediated by these cells [107]. Inflammatory conditions and elevated insulin levels may enhance the expression of CD26/DPP4 on adipocyte surfaces and its subsequent release into the bloodstream [26, 108].

Only a single study has examined the serum levels of CD26/DPP4 in AD patients. The findings revealed that circulating CD26/DPP4 levels have no difference between AD and control groups [109]. Another study investigated the expression of CD26/DPP4 on cell surfaces, revealing its minimal presence in the epidermis of healthy individuals. However, increased expression was observed in the lesioned skin of AD patients [110]. In order to get a greater comprehension of the role of CD26/DPP4 in the inflammation of the skin, researchers generated rats lack of CD26/DPP4 for experimental purposes. Rats deficient in these genes demonstrated a significant elevation in activated Th cells and a reduction in Treg cells in their peripheral blood compared with their wild-type counterparts. In a dermatitis model induced by 1-chloro-2,4-dinitrochlorobenzene, representing Th1-like inflammation, the ears of CD26/DPP4-deficient rats exhibited more pronounced thickening compared with those of wild-type rats. The skin lesions in these rats exhibited a significant increase in the concentrations of IL-4, IL-6, IL-10, and IFNγ. Conversely, wild-type rats exhibited more severe dermatitis in a Th2-type skin inflammation model triggered by toluene-2,4-diisocyanate. Additionally, CD26/DPP4-deficient rats exhibited decreased levels of IFNγ and IL-6. Interestingly, there was a significant upregulation of Treg cells in wild-type rats following induction but not in CD26/DPP4-deficient rats [110]. These findings suggest that CD26/DPP4 may enhance inflammation via the Th2-type pathway and suppress the Th1 inflammatory pathway.

### Other adipokines

There are nine fatty acid binding proteins (FABP) with specific expression patterns in various tissues or organs. Adipocyte-FABP (FABP4) is predominantly expressed in adipocytes and macrophages, both of which are implicated in metabolic disorders, cardiovascular diseases, neoplasms, and asthma. Epidermal-FABP (FABP5) is ubiquitously expressed in the skin, adipocytes, and macrophages. Abnormalities in this context can lead to inflammatory skin diseases, systemic inflammation, and asthma [111]. Elevated levels of serum FABP4 have been found in children with AD, whereas the expression levels of the FABP5 gene are notably greater in AD lesions compared to normal skin. Furthermore, a positive correlation between these levels and the severity of the lesions is observed [112–114]. Additionally, a positive association was identified between the levels of circulating FABP5 and total IgE [114].

Apelin is widely distributed in adipose tissues, kidneys, nervous system, and other tissues. The active form of apelin comprises peptides of varying lengths produced by the cleavage of the preprotein. Different isoforms of apelin bind to specific receptors to perform distinct functions. Apelin has been definitively implicated in apoptosis, inflammation, glucose metabolism, and immunomodulation [115]. Insulin resistance enhances apelin secretion by adipocytes, subsequently forming a negative cycle of feedback in the process of insulin secretion [116]. An investigation found that apelin was significantly increased in children with AD, with a notable sex disparity. Specifically, apelin concentrations in the female cohort were significantly higher than those in the male cohort across both AD and control groups [99].

Zinc-α2-glycoprotein (ZAG) participates in lipid metabolism [117]. ZAG, acting as an adipose mobilizing factor, is regulated by growth hormones to stimulate lipolysis and demonstrates anti-inflammatory properties [118]. Accumulation of fat reduces serum levels of ZAG [119]. Patients with AD have a reduction of the mRNA expressed by AZGP1 gene and ZAG protein levels in the stratum corneum. Simultaneously, circulating levels of ZAG were decreased, with no significant correlation observed with disease severity. To further elucidate the pertinent molecular mechanisms, supplementary in vitro experiments were conducted on human epidermal keratinocytes. The findings revealed that suppressing the AZGP1 gene resulted in a reduction of FLG and an elevation of TSLP. The corresponding suppression of FLG expression and exposure to TSLP could also result in a reduction of ZAG expression [120]. This implies that ZAG can modulate both FLG and TSLP and can also be modulated by them. It was also found that the topical application of ZAG to an AD mouse model facilitated skin barrier repair by regulating ceramide synthesis. However, the skin of a normal mouse remained unaffected by either ZAG or ZAG monoclonal antibody treatment. At the molecular level, the assay demonstrated that treatment with ZAG reduced IL-4, IL-17a, and IFN-γ while increasing Foxp3 in mice.

## Discussion

Numerous studies have demonstrated that abnormal expression of leptin, adipokines, resistin, visfatin, DPP4, FABP, apelin, and ZAG is observed in individuals diagnosed with AD. The comprehensive alterations in these adipokines in patients with AD are detailed in Table 1. Similar patterns of adipokine abnormalities can be observed in both AD and obesity, reinforcing the association between the two.

**Table 1:** Results of existing studies concerning the changes of adipokines in AD patients

Adipokine	Study	Subjects	Sample size		Change In AD	Relationship With Severity of AD^a^	Correlation With IgE
			**Patients with AD**	**Control**			
Leptin	Jung MJ et al., Korea, 2020 [20]	Adults	40	6	Increase in circulation	No correlation (EASI)	Positive
	Kimata H, Japan, 2002 [54]	Children	25	25	Increase in circulation	UN	Positive
	Jaworek AK et al., Poland, 2020 [55]	Adults	49	30	Increase in circulation	No difference between mild and severe eczema^b^	No correlation
	Kimata H, Japan, 2004 [56]	Lactating women	30	30	Decrease in circulation	UN	UN
	Balato N et al., Italy, 2011 [57]	Teenagers^e^ and adults	5	117	Decrease in circulation	UN	UN
	Bostanci I et al,. Turkey, 2004 [60]	Children	20	20	No difference	UN	UN
	Seo S et al., Korea, 2016 [58]	Children	59	31	Decrease in circulation	No correlation (SCORD); mild group higher than severe group^c^	UN
	Han B et al., Korea 2016 [59]	Children and adults	64	UN	UN	No correlation (EASI)	UN
	Banihani SA et al., Jordan, 2018 [61]	Children and adults	164	167	UN	UN	UN
Adiponectin	Jung MJ et al., Korea, 2020 [20]	Adults	40	6	Decrease in circulation	No correlation (EASI)	No correlation
	Jaworek AK et al., Poland, 2020 [55]	Adults	49	30	Decrease in circulation	No difference between mild and severe eczema^b^	No correlation
	Seo S et al., Korea, 2016 [58]	Children	59	31	No difference	No correlation (SCORD); No difference between mild and severe group^c^	UN
	Han B et al., Korea 2016 [59]	Children and adults	64	UN	UN	No correlation (EASI)	UN
	Al-Shaheri F et al., Jordan, 2022 [76]	Children and adults	162	162	Decrease in circulation	UN	UN
	Lee SH et al., Korea, 2022 [77]	Adults	75	28	Decrease in circulation	Negative (EASI)	No correlation
	Nagel G et al., Germany, 2009 [78]	Children	50	338	UN	UN	UN
	Seo HS et al., Korea, 2019 [79]	In vitro epidermal equivalent model	UN	UN	UN	UN	UN
Resistin	Jaworek AK et al., Poland, 2020 [55]	Adults	49	30	Decrease in circulation	Negative (SCORAD)	Negative
	Banihani SA et al., Jordan, 2018 [97]	Children and adults	162	161	Decrease in circulation	UN	UN
	Farag AGA et al., Egypt, 2020 [98]	Children and adults	45	40	Decrease in circulation	Negative (SCORAD)	UN
	Machura E et al., Poland, 2013 [99]	Children	27	46	Increase in circulation	UN	UN
Visfatin	Suga H et al., Japan, 2013 [104]	Adults	40	42	Increase in circulation	No difference between mild, moderate and severe eczema^d^	UN
	Machura E et al., Poland, 2013 [99]	Children	27	46	Decrease in circulation	UN	UN
DPP4	Miyagaki T et al., Japan, 2013 [109]	Adults	32	27	No difference	No correlation (Rajka Langeland severity score)	No correlation
	Tasic T et al., Germany, 2011 [110]	Skin biopsies and rats	UN	UN	UN	UN	UN
FABP4	Brunner PM et al., USA, 2019 [112]	Children and adults	88	37	Increase in circulation^g^	UN	UN
FABP5	Takahashi-Shishido N et al., Japan, 2021 [113]	Skin biopsies	12	8	Increase in skin	UN	UN
	Yamane Y et al., Japan, 2009 [114]	Horny layer samples	36	16	Increase in circulation skin	Positive (local severity score^h^)	UN
Apelin	Machura E et al., Poland, 2013 [99]	Children	27	46	Increase in circulation	UN	UN
ZAG	Noh JY et al., Korea, 2019 [120]	Patients with AD^f^, skin biopsies and rats	90	36	Decrease in circulation	No correlation (EASI)	UN

*Abbreviations: AD Atopic dermatitis, DPP4* Dipeptidyl peptidase-4*, EASI* Eczema Area and Severity Index*, FABP* Fatty acid binding protein*, IgE* Immunoglobulin E*, SCORAD* SCORing Atopic Dermatitis index*, UN Unmentioned, ZAG Zinc-α2-glycoprotein*

^a^In correlation analysis, severity of AD was quantified by different methods given in parentheses

^b^*mild: SCORAD* < *25; severe: SCORAD* > *50*

^c^*mild: SCORAD* < *40; severe: SCORAD* ≥ *40*

^d^*identified by Rajka Langeland severity score*

^e^*Teenagers are defined as minors older than 14*

^f^*Ages of AD patients are not noted*

^g^*only children show significant increase of FABP4 in AD*

^h^*Local severity score of a measured lesion was presented by the sum of the respective severity scores (0–24) based on the modified scoring scale of each eruption shown in the SCORAD index and clinical features shown by Leung*

However, it has also been established that obesity is associated with some inflammatory conditions, such as psoriasis, a Th7-mediated inflammatory disease. Recent studies have considered adipokines as a critical link between these two diseases. While AD is a Th2-mediated disease, obesity facilitates two inflammatory skin diseases with distinct immune pathways by abnormal adipokines.

**Fig. 2:**
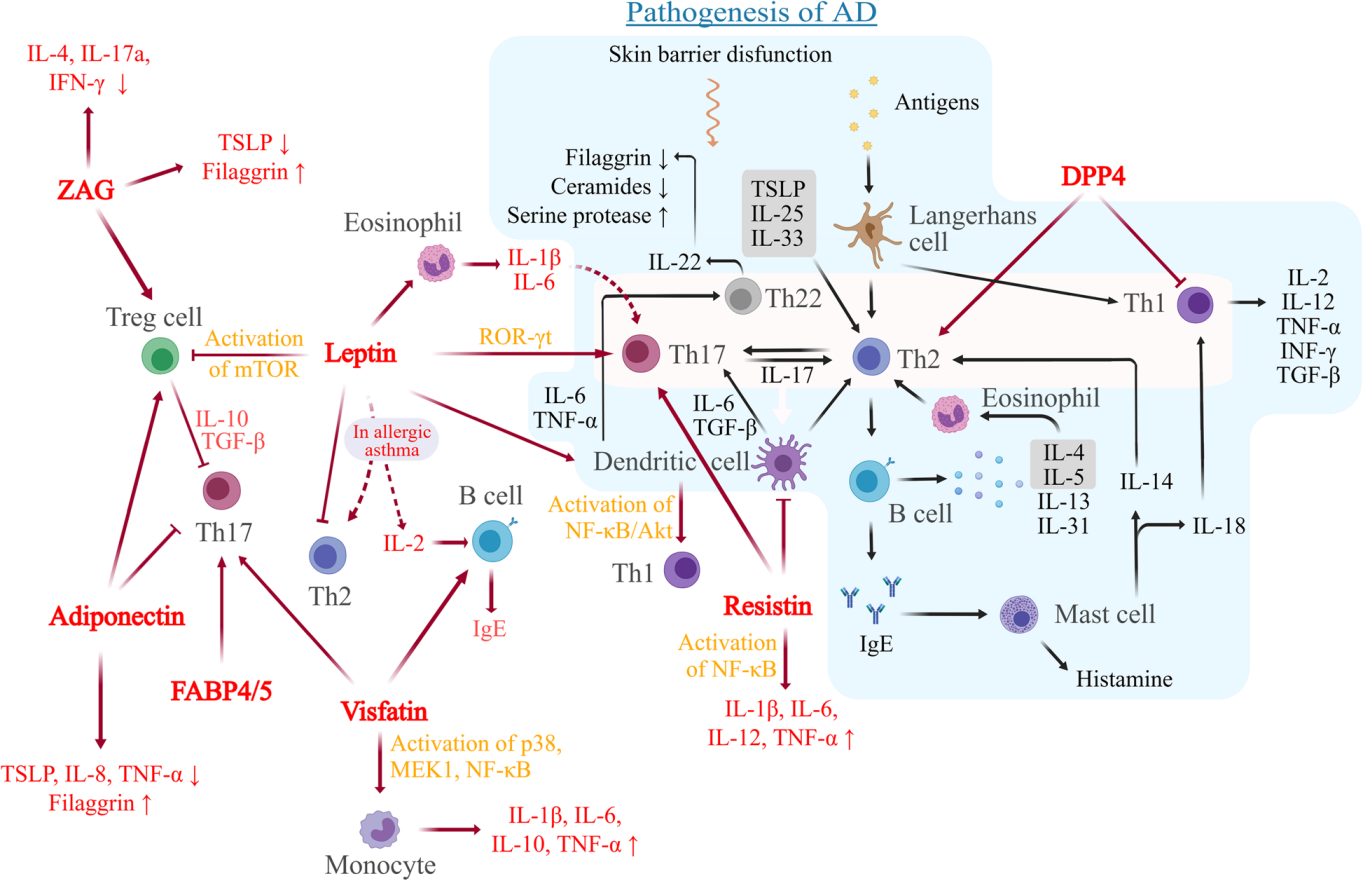
Roles of adipokines in AD pathogenesis. Adipokines are known to influence the regulation of immune and inflammatory pathways, thus contributing to the advancement of AD. AD, atopic dermatitis; Th, helper T; TSLP, thymic stromal lymphopoietin; IL, interleukin; TNF, tumor necrosis factor; INF, interferon; TGF, transforming growth factor; Treg cell, regulatory T cell; ROR, retinoic acid-related orphan receptor; mTOR, mammalian target of rapamycin; NF-κB, nuclear factor-kappa beta; MEK1, mitogen-activated proteinkinase kinase 1; FABP, fatty acid binding protein; ZAG, zinc-α2-glycoprotein; DPP4, dipeptidyl peptidase 4

Figure 2 depicts how adipokines may contribute to AD pathogenesis. While some studies suggest that dysregulated adipokines may stimulate Th2 response, adipokine patterns in AD focus more on inducing Th17 responses [121]. This finding contradicts the Th2-dominant pathogenesis of AD. In fact, Th17 may be the dominant factor in obesity-related AD. In obese mice, T cells with IL-4 and IL-13 increased by approximately 3.9- and 1.7-fold, respectively. However, T cells with IL-17A and IL-17F increased by 6.5- and 11.5-fold, respectively. In patients with AD, markers of Th17 were positively correlated with BMI [122]. These results indicate that obesity may alert the predominant mechanism of AD towards the Th17 response and ultimately exacerbate the disease. T cells have peroxisome proliferator-activated receptor-γ (PPARγ), which induces a Th2-predominant state and helps prevent other types of inflammation. However, decreased activity of PPARγ in Th2 cells was observed in obese mice, and treatment with PPARγ agonist helped limit the activation of Th17. Additionally, PPARγ agonist aided in improving the effect of anti-IL-4/IL-13 treatment for AD in obese mice [122]. Adipokines may facilitate this kind of Th17-dominant AD in individuals who are obese. In individuals with AD, abnormal adipokines are probably part of the Th17 pathway, which in turn can worsen AD and increase the risk of obesity. The precise function of Th2 in AD is not yet fully understood.

There is indirect evidence suggesting that Th17 cells have a more prominent function in obesity-related AD. According to the findings, there is a more consistent association between AD and obesity in younger children [12]. Meanwhile, early onset of AD and children with AD are correspondingly associated with stronger Th17-skewing [39, 43]. It may be necessary to pay more attention to weight management to prevent AD in younger children.

## Strengths and limitations

Dysregulated adipokines in patients with AD have not been summarized before. This review reveals the possible functions of adipokines in the development of AD. It provides a new perspective on AD pathogenesis and is conducive to understanding the association between AD and obesity. Additionally, adipokines could be new therapeutic targets to improve AD treatment for patients who have poor responses to conventional treatments. As a straightforward method for restoring adipokine levels to their normal range, reducing body weight has been demonstrated to reduce the severity of AD. Consequently, weight reduction could likely be incorporated into the management of AD.

However, there are some limitations to consider. Different studies have reported inconsistent results concerning the same adipokine. The observed discrepancy may arise from the heterogeneous nature of AD. Various phenotypes of AD have been described with distinct pathogenesis, which may act as confounding factors. The characteristics of study populations, including nutritional status, sex disparities, and racial diversities, can also impact adipokine conditions [123–125]. It should be noted that the studies on AD did not follow the same diagnostic criteria and used different laboratory methods to measure adipokines. This variation in sample collection time points might have introduced biases. Additionally, some studies did not document the treatments received by patients, which could be potential confounding factors.

Establishing a definitive causal relationship between AD and dysregulated adipokines presents a significant challenge in cross-sectional studies. The relationships among adipokine serum concentrations, disease severity, and total IgE levels remain complex and unclear. To clarify the adipokine alterations and functions of adipokines in AD, large-scale clinical trials, prospective studies, and mechanistic research are necessary.

## Conclusions

In summary, the dysregulation of specific adipokines exhibits a strong correlation with AD. The investigation of adipokines in AD patients enriches the comprehensive understanding of AD pathogenesis. Animal and in vitro cellular studies can offer partial elucidation of the intricate pathways through which adipokines may contribute to AD pathogenesis. Given the rising prevalence of obesity, it is imperative to explore the potential association between AD and obesity. The potential role of adipokines as critical mediators connecting obesity and AD is emphasized. With the growing comprehension of their roles in AD, adipokines have emerged as promising targets for AD therapy. Adipokine-targeted therapy is supposed to effectively treat AD patients with inadequate response to current treatments.

## Data Availability

Not applicable.

## Abbreviations

AD: Atopic dermatitis
ORs: Odds ratios
BMI: Body mass index
IL: Interleukin
TNF: Tumor necrosis factor
IgE: Immunoglobulin E
JAK: Janus kinase
FLG: Filaggrin
IFN: Interferon
Treg: Regulatory T
SCORAD: SCORing Atopic Dermatitis Index
EASI: Eczema Area and Severity Index
ROR: Retinoic acid-related orphan receptor
mTOR: Mammalian target of rapamycin
SNPs: Single nucleotide polymorphisms
AdipoR: Adiponectin receptor
TSLP: Thymic stromal lymphopoietin
NF-κB: Nuclear factor-kappa beta
DCs: Dendritic cells
NAMPT: Nicotinamide phosphoribosyltransferase
NAD: Nicotinamide adenine dinucleotide
FABP: Fatty acid binding protein
ZAG: Zinc-α2-glycoprotein
PPARγ: Peroxisome proliferator-activated receptor-γ
